# Potential Mechanisms Connecting Purine Metabolism and Cancer Therapy

**DOI:** 10.3389/fimmu.2018.01697

**Published:** 2018-07-30

**Authors:** Jie Yin, Wenkai Ren, Xingguo Huang, Jinping Deng, Tiejun Li, Yulong Yin

**Affiliations:** ^1^Guangdong Provincial Key Laboratory of Animal Nutrition Control, College of Animal Science, Institute of Subtropical Animal Nutrition and Feed, South China Agricultural University, Guangzhou, China; ^2^Scientific Observing and Experimental Station of Animal Nutrition and Feed Science in South-Central, Ministry of Agriculture, Hunan Provincial Engineering Research Center of Healthy Livestock, Key Laboratory of Agro-Ecological Processes in Subtropical Region, Institute of Subtropical Agriculture, Chinese Academy of Sciences, Changsha, China; ^3^University of Chinese Academy of Sciences, Beijing, China; ^4^Department of Animal Science, Hunan Agriculture University, Changsha, Hunan, China

**Keywords:** purine, purinosome, metabolism, cancers, mammalian target of rapamycin

## Abstract

Unrestricted cell proliferation is a hallmark of cancer. Purines are basic components of nucleotides in cell proliferation, thus impaired purine metabolism is associated with the progression of cancer. The *de novo* biosynthesis of purine depends on six enzymes to catalyze the conversion of phosphoribosylpyrophosphate to inosine 5′-monophosphate. These enzymes cluster around mitochondria and microtubules to form purinosome, which is a multi-enzyme complex involved in *de novo* purine biosynthesis and purine nucleotides requirement. In this review, we highlighted the purine metabolism and purinosome biology with emphasis on the therapeutic potential of manipulating of purine metabolism or purinosome in cancers. We also reviewed current advances in our understanding of mammalian target of rapamycin for regulating purinosome formation or purine metabolism in cancers and discussed the future prospects for targeting purinosome to treat cancers.

## Introduction

Normal cells undergo a series of highly regulated physiological responses to provide necessary substrates for the basic cellular processes, while cancer cells are involved in a complex metabolic rearrangement characterized by an increase in energy production and biosynthetic processes to sustain cell growth and proliferation (1–8). Purines are the most abundant metabolic substrates for all living organisms by providing essential components for DNA and RNA. Besides as building blocks for DNA and RNA, purines provide the necessary energy and cofactors to promote cell survival and proliferation. Thus, purines and their derivatives widely participate in biological processes, including immune responses and host–tumor interaction (9). Notably, high concentrations of purine metabolites have been indicated in tumor cells, and this discovery favors to the development of the earliest antitumor drugs (purine antimetabolites) to treat cancers by blocking DNA synthesis and halting cell growth. Purinosome has been recently identified within purine metabolism, and the formation of purinosome is closely related to the cell cycle (10, 11). These results provide a novel therapeutic strategy for cancers by targeting purinosome formation and purine metabolism.

In this review, we discuss the purine metabolism, including the complementary salvage pathway and *de novo* biosynthetic pathway. We then discussed the purinosome with emphasis on purinosome formation and composition, and its interaction with mitochondria. We also described the potential therapeutic strategies for cancers by targeting purine metabolism and purinosome, which may be used to reprogram cancer metabolism. Finally, the mechanism of mammalian target of rapamycin (mTOR) regulating the formation of purinosome is discussed.

## Purine Metabolism

**Figure 1:**
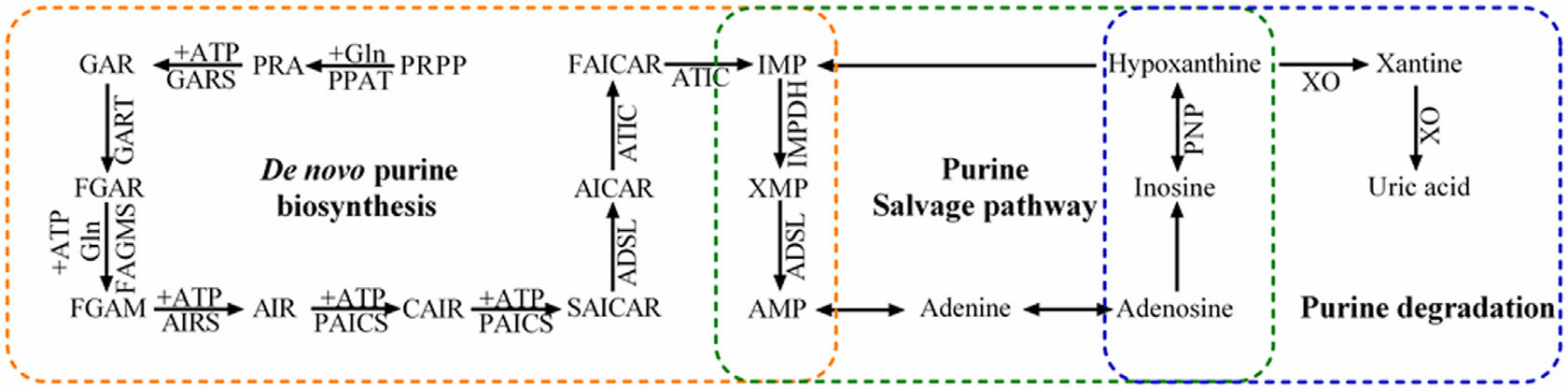
Purine metabolism pathways. Purine metabolism includes *de novo* purine biosynthetic pathway, purine salvage pathway, and degradation. The *de novo* purine biosynthetic pathway uses six enzymes to catalyze the transformation of phosphoribosylpyrophosphate (PRPP) into inosine 5′-monophosphate (IMP) *via* 10 highly conserved steps (orange). Purine salvage (green) recycles hypoxanthine, inosine, and adenine as substrates to generate purine nucleotides. Inosine and hypoxanthine can be further oxidized into xanthine and uric acid in the purine degradation pathway (blue).

Purine metabolism maintains cellular pools of adenylate and guanylate *via* synthesis and degradation of purine nucleotides. In mammalian cells, purine nucleotides are synthesized in two different pathways: the complementary salvage pathway and *de novo* biosynthetic pathway (Figure 1). Generally, the complementary salvage pathway accounts for most of the cellular requirements for purine by recycling the degraded bases with help of hypoxanthine-guanine phosphoribosyltransferase (HPRT) and adenine phosphoribosyltransferase (Figure 1). HPRT is an Mg_2_^+^-dependent enzyme and recycles hypoxanthine and guanine *via* transferring phosphoribosyl group from phosphoribosylpyrophosphate (PRPP) to generate inosine monophosphate (IMP) and guanine monophosphate (GMP), respectively.

Under the conditions with higher requirement for purine nucleotides, such as dividing cells and tumor cells, the *de novo* biosynthetic pathway is fundamental to replenish the purine pool. In humans, six enzymes catalyze PRPP to form IMP in 10 highly regulated and conserved steps. These enzymes include one trifunctional enzyme (TrifGART: GARS, GART, and AIRS domains), two bifunctional enzymes (PAICS: CAIRS and SAICARS domains; ATIC: AICART and IMPCH domains), and three monofunctional enzymes (PPAT, FGAMS, and ASL). The generated IMP contributes to the production of various intermediates, such as AMP, GMP, adenosine, and inosine. Inosine is further converted to hypoxanthine by purine nucleoside phosphorylase (PNP), and xanthine-oxidase (XO) catalyzes hypoxanthine oxidation to form xanthine (12).

The *de novo* biosynthetic pathway is energy intensive, and numerous amino acid substrates and one-carbon units contribute to the 10-step enzymatic processes, such as glutamine, ATP, and formate. In this pathway, five molecules of ATP, two molecules of glutamine and formate, and one molecule of glycine, aspartate, and carbon dioxide are necessary for generation of one molecule of IMP (13). Therefore, these substrates play a critical role in purine metabolism especially in rapid proliferating cancer cells. Indeed, metabolic dependencies on glutamine and aspartate are increased to fuel anabolic processes to support cancer growth (14), and glycine metabolism contributes to biosynthetic requirement of purines, ATP, and NADPH in cancer cells (15).

## Purinosome

Metabolic pathway is generally assembled with several sequential enzymes into a higher order protein structure to facilitate metabolic flux, and the formation of the protein complex of enzymes is known as metabolon (16). Metabolon has been found in various metabolic pathways, such as the glycosome in the glycolytic pathway (17). Metabolon improves the efficiency of metabolic pathway by increasing the local concentration of intermediates and metabolic substrates, decreasing the concentration of enzymes needed to maintain a given flux, directing the products to a specific subcellular location, or minimizing the escape of reactive intermediates (16, 18). For example, compared to the effector T cells, which have more “fissed” mitochondria, memory T cells have more “fused” mitochondria to densely pack the electron transport chain complexes to form respirasomes, resulting in efficient transfer of electrons and minimizing proton leak during ATP production (19). Purine metabolism has been investigated for decades and there is a wide speculation that enzymes in the *de novo* purine biosynthesis are organized into a metabolon to maintain cellular purine pool. In 2008, purinosome was first confirmed in living cells through the discovery that all six enzymes in the *de novo* biosynthetic pathway are recruited to form punctate bodies in the cellular cytoplasm (10). The assembly and disassembly of purinosome can be regulated dynamically by cellular level of purine (10, 20), which directly activates the *de novo* purine biosynthesis (21, 22).

During cell growth or division, the *de novo* purine biosynthesis is significantly activated to provide purine nucleotides for growth (G1), duplication of genetic materials (S), division (G2), and divide (M) (23–25). Through the time-lapse fluorescence microscopy assay, high percent of purinosome-positive cells are identified in the G1 phase of cell cycle when purine demand is the highest (11). The requirement of purine decreases with progression of cells into the S and G2/M phases, which accounts for the drop in the relative amount of purinosome-positive cells throughout the cell cycle (11).

**Figure 2:**
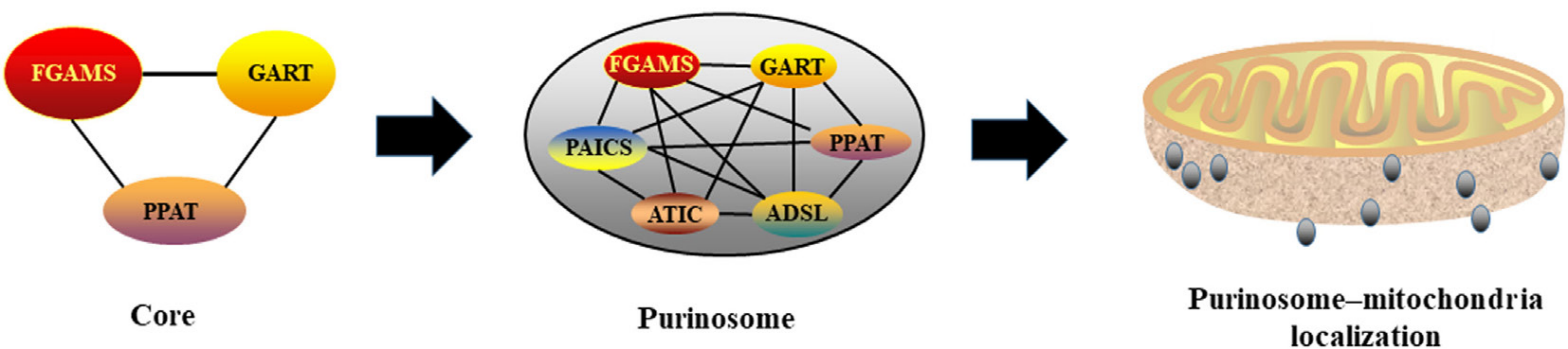
Purinosome formation and its interaction with mitochondria. PPAT, GART, and FGAMS constitute the core scaffolding structure of purionsome, then, PAICS, ADSL, and ATIC appear to interact peripherally. Assembled purionsomes are mainly localized with mitochondria and interact mutually.

Through mapping protein–protein interactions within the purinosome, Deng et al. found that the first three enzymes in the pathway constitute the core scaffolding structure, including PPAT, GART, and FGAMS, whereas PAICS, ADSL, and ATIC appear to interact peripherally (26) (Figure 2). Deficiency or mutation of any enzymes within purinosome impairs purinosome formation and complex stability (27, 28). Purine supplementation or deficient in specific enzymes results either in a complete loss or a significant reduction of purinosomes (13). Various factors have been identified to affect purinosome function. For example, mitochondrial tetrahydrofolate (THF), an essential substrate for *de novo* purine synthesis, enhances purine biosynthesis by delivering 10-formyl THF to purinosome (29). Interestingly, although purinosome-positive cells vary from G1 to G2/M phases, the expressions of enzymes in the *de novo* purine biosynthetic pathway are not altered throughout the cell cycle (11). Therefore, we anticipate that the assembly and disassembly of purinosome in cells are not totally governed by protein abundances.

Considering that five molecules of ATP are needed for generating one molecule of IMP, mitochondrial function may be associated with purinosome biology and *de novo* purine biosynthesis. Indeed, mitochondrial THF cycle contributes to non-essential amino acids and one-carbon formyl units to mediate production of purine nucleotides (30). Using two-color 3D STORM imaging, a substantial larger fraction of purinosomes is localized with mitochondria rather than randomly distributed within the cytoplasm in purinosome-positive cells (31), indicating a high possibility of ordered distribution of purinosomes and mitochondria (Figure 2). This is further supported by the discovery that purinosome proteins (i.e., ASL and FGAMS) are co-precipitated with the purified mitochondria, indicating a potential possibility of interaction between mitochondria and purinosome. Mitochondria dysregulation (i.e., defect in electron transport or oxidative phosphorylation) enhances purinosome formation in cells and inhibition of purinosome formation also impairs mitochondrial metabolism in purine-rich conditions (31). Although the molecular mechanisms of the purinosome and mitochondria interaction still need to be uncovered, mitochondria provides enough ATP, amino acid substrates, and one carbon formyl units for the *de novo* purine biosynthesis, which partially helps to explain the potential mechanism (13).

## Purine Metabolism in Cancers

Aberrant cell cycle with uncontrolled cell proliferation is a hallmark of cancers, thus targeting cell cycle has been considered as an attractive strategy for cancer therapy (32–38). Purines and enzymes for *de novo* purine biosynthetic pathway are enhanced in tumor cells because purine nucleotides are fundamental and necessary for tumor cell proliferation (39–42). For example, inosine strongly enhances proliferation of human melanoma cells (43), and altered ratio of adenosine to inosine has been widely noticed in cancer cells, affecting growth, invasiveness, and metastasis (44, 45). Meanwhile, purines serve as potent modulators in the response of immune cells and cytokine release *via* various receptor subtypes, such as P2X ligand-gated ion channels and G protein-coupled P2Y receptors (46, 47), which is substantially involved in the development of oncogenesis and tumorigenesis (48–51). For example, adenosine plays a role in the regulation of neutrophil function and modulates the interaction of neutrophils with pathogens (52). Also, mutation or deficiency of adenosine deaminase, which is a key enzyme for purine metabolite degradation or salvage into the nucleotide pool, increases susceptibility to infections and autoimmunity, and adenosine deaminase activity has been used as a diagnostic marker for cancers (53, 54). More specific details about the interaction between purine metabolism and immune system in tumor microenvironment can be found in reviews by Antonioli et al. (55), Kumar (56), and Muller-Haegele et al. (57). Therefore, targeting purine metabolism may serve as a potential therapy in cancers.

Aberrant metabolisms of amino acids and one-carbon units for purine metabolism are also presented in cancer cells, which can be used to predict the subtype of cancer and disease progression (58). For example, glutamine is a key substrate for catalytic activity of PPAT and FGAMS in the purinosome and plays important roles in anabolic processes and physiological responses in cancer cells, including sustaining proliferative signaling, enabling replicative immortality, resisting cell death, and invasion and metastasis (14, 59). Moreover, other substrates of purine metabolism, such as glycine, aspartate, and precursor of one-carbon unit, have also been reported to anticipate in the rapid cancer cell proliferation (60–64), thus, targeting amino acid substrates or one-carbon unit metabolism may serve as a potential therapeutic power of manipulating cell proliferation for treating cancers. Indeed, targeting glutamine metabolism and uptake in cancer cells reduces tumor weight, nodules, and metastasis (65, 66). One-carbon unit also support the high proliferative rate of cancer cells, thus, antifolate drugs that target one-carbon metabolism have long been used in the treatment of cancers. For example, cancer cells are particularly susceptible to deprivation of one-carbon units by serine restriction or inhibition of *de novo* serine synthesis (67). Also, dietary starvation of serine or glycine reduces tumor growth and improves survival in different cancer models through antioxidant responses and mitochondrial oxidative phosphorylation (68, 69).

Purine antimetabolites are the one of earliest developed chemotherapy drugs and have been widely used in the clinic to treat cancers (70, 71). Currently, more than 10 purine antimetabolites have been approved by the Food and Drug Administration for treatment of cancers, such as 6-mercaptopurine, 6-thioguanine, and methotrexate. Purine antimetabolites are chemical analogs sharing with similar structure to the metabolites in the purine metabolism and can compete to incorporate into purine nucleotides and DNA during the S phase of the cell cycle to inhibit rapid division and proliferation (72, 73). Unfortunately, resistance of antimetabolites often occurs, and various genes are involved in the intolerance (74, 75). In addition, purine antimetabolites also affect the proliferation of healthy cells and thereby cause potential toxicity to normal cells. Therefore, it is urgent to identify new regulatory targets within purine metabolism, which would inhibit tumorigenesis without drug resistance and hurting normal cells.

Targeting purine degradation has also been used to treat cancers as enhanced purine degradation limits the available purines for nucleotides synthesis, resulting in inhibition of cell proliferation in cancers. For example, an intratumoral injection of adenoviral vector expressing *E. coli* PNP to accelerate inosine degradation shows safety and antitumor activity in the first-in-human clinical trial (76). Activation of XO suppresses disulfide bond formation of breast cancer resistance protein (77), and reactive oxygen species derived from XO further interrupt dimerization of breast cancer resistance protein (78, 79).

Considering that purinosome formation plays an important role in *de novo* purine biosynthesis, one strategy may arise by targeting purinosome assembly/disassembly in cancers. For example, G protein-coupled receptors (GPCRs) regulate a myriad of biological responses *via* multiple signaling pathways in both normal and cancer cells (80–82). GPCRs have also been demonstrated to affect purinosome assembly/disassembly to control metabolic flux *via de novo* purine biosynthesis in human cancer cells (83, 84), which may contribute to the regulatory mechanism of GPCRs in cancers. Heat shock protein 90 (Hsp90) and Hsp70 functionally colocalize with purinosomes (85), thus inhibitors of Hsp90 and Hsp70 reversibly disrupt purinosome formation, and have a synergistic effect with methotrexate (86) to treat cancers (87, 88). Most cancer cells abundantly express Hsp90/Hsp70 (89), and its chaperone machinery in the assembly of the purinosome may provide a novel strategy for the development of advanced anticancer therapies *via* disrupting purine biosynthesis. Upregulations of specific pathway enzymes (i.e., PPAT, PAICS, and ATIC) in numerous cancers indicate an importance of purine metabolism and purinosomes in tumorigenesis (41, 90), while mutations of these enzymes affect purinosome assembly in cultured skin fibroblasts from patients with AICA-ribosiduria and ADSL deficiency (27). In conclusion, disruption of purinosome formation is likely to mediate cell cycle and to enhance sensitivity to cancer chemotherapeutics (86).

## mTOR-Mediated-Purinosome and Purine Metabolism in Cancers

Purinosomes and mitochondria interaction may improve the efficiency of *de novo* purine biosynthesis, thus disruption in the purinosome juxtaposition to the mitochondria may serve as a novel therapeutic potential for cancers. Using human kinome screen, mTOR has been identified as a putative kinase network associated with the translation of chemical signals into purinosome and mitochondria interaction (31). In sporadic cancers, mTOR activation is the result of amplification/activation mutations in genes encoding upstream tumor signal transduction cascades or deletion/inactivation of tumor suppressors (91–93). In response to proliferating signal in cancer cells, mTOR activates ATF4, which stimulates the expressions of MTHFD2 and other enzymes for serine synthesis and THF cycle, providing amino acid substrates and one-carbon units required for the *de novo* purine synthesis (30). Rapamycin (a mTOR inhibitor) inhibits the interaction between purinosomes and mitochondria in a dose-dependent manner (31). Mitochondria dysregulation enhances the formation of purinosomes and the percentage of purinosome-positive cells, while these increases are abrogated by rapamycin (31). Meanwhile, mTOR is also suppressed by a *de novo* purine synthesis antagonist (AG2037) that reduce intracellular purine nucleotide pools *via* reducing the level of GTP-bound Rheb, an obligate upstream activator of mTOR complex 1 (mTORC1) (94). Meanwhile, AG2037 treatment markedly inhibits mTORC1 activation and robust tumor growth in mice bearing non-small-cell lung cancer xenografts (94). These results confirm the mutual interaction among mTOR, mitochondria, and the *de novo* purine biosynthetic pathway, and further studies are needed to explore the therapeutic effects of inhibitors for purine biosynthesis and the underlying mechanism of mTOR-mediated purinosome-mitochondria localization and purine metabolism in cancers.

**Figure 3:**
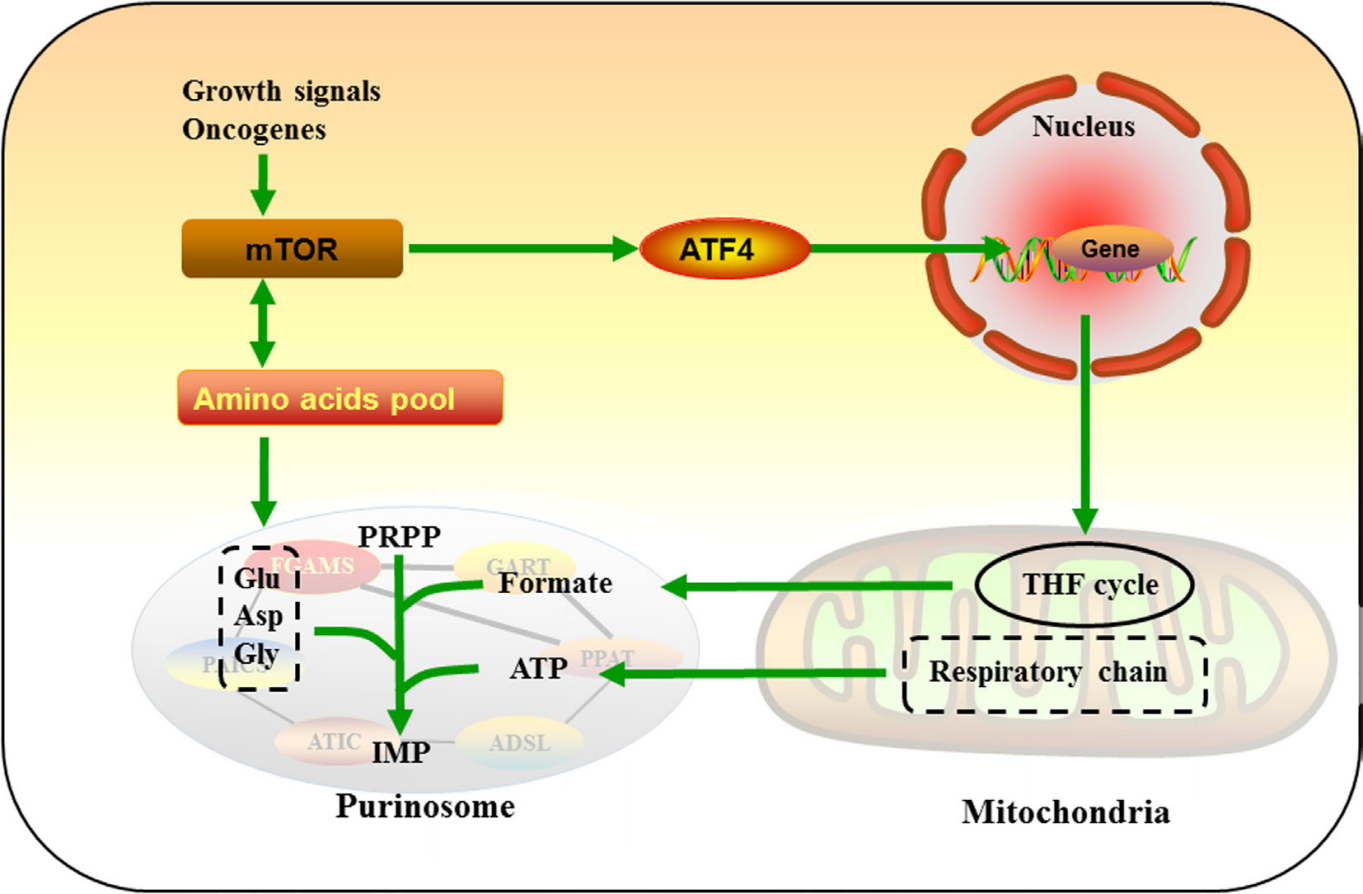
Mammalian target of rapamycin (mTOR)-mediated purine metabolism. In response to growth signal in cancer cells, mTOR activates ATF4, which upregulates enzymes of tetrahydrofolate cycle and provides one-carbon unit formate for *de novo* purine synthesis. mTOR also serve as a master regulator that senses and mediates amino acids pool, which may further regulate glutamine (Gln), glycine (Gly), and aspartate (Asp) flux into purinosomes.

Recent work has shown that alterations of amino acid metabolism are common observed in cancers, and glutamine is an abundant and versatile nutrient that participates in the growth and metabolism of cancer cells (95–97). Glutamine also serves as a substrate of PPAT and FGAMS for catalyzing PRA and FGAM formation in the *de novo* purine biosynthesis, which may partially explain why glutamine is one of the most highly consumed nutrient by cancer cells. mTOR also serve as a master regulator that senses amino acid availability to regulate cell growth in normal and cancer cells (98–104). For example, glutamine transportation plays a key role in controlling cellular metabolism, growth, and survival *via* mTOR signal in lung cancer and breast cancer (105, 106). Also, mTOR-mediated alteration of amino acid substrates (glycine and aspartate) for the *de novo* purine biosynthesis has been reported in a subset of tumors (107–109). Collectively, these studies suggest that mTOR is a critical regulatory signal in the *de novo* purine biosynthetic pathway by influencing purinosome and mitochondria interaction and amino acid substrates, which further reprogram metabolic responses in cancer cells (Figure 3).

## Conclusion and Future Perspectives

Purines are the scaffold substrates of nucleic acids, coenzymes, allosteric modulators, and energy intermediates for cells. Thus, purine metabolism is associated with several of biochemical reactions, including metabolism, cell cycle, immune function, and signal transduction. Recently, scientists have identified purinosomes, which formed from the *de novo* purine biosynthesis enzymes to improve the efficiency of metabolic flux in the cells when purines are highly required. In addition to providing ATP for the *de novo* purine biosynthesis, there may be a potential interaction between mitochondria and purinosome, which further accelerates purine synthesis. Several decades ago, targeting purine metabolism has been used to design the antimetabolite drugs to treat cancers, and purinosome represents a novel target to pharmacologically control cellular metabolism.

Although the current results are inspiring, various questions have been raised after the discovery and characterization of the purinosome. For example, purinosome-mitochondria colocalization plays an important role in purine metabolism, how does purinosome sense and locate mitochondria? Besides mTOR-mediated link between purinosomes and mitochondria, is there any other signaling events involving purinosome formation? Protein-coupled receptors and p38MAPK have been reported to activate purine metabolism to initiate cell cycle in response to stress (25, 83, 110), the mediatory roles of these signals in purinosome need to be further studied.

## Author Contributions

JY wrote the manuscript. XH, JD, TL, and YY discussed the topic. WR and XH revised the manuscript.

## Conflict of Interest Statement

The authors declare that the research was conducted in the absence of any commercial or financial relationships that could be construed as a potential conflict of interest.

## Abbreviations

mTOR, mammalian target of rapamycin; HPRT, hypoxanthine-guanine phosphoribosyltransferase; APRT, adenine phosphoribosyltransferase; PRPP, phosphoribosylpyrophosphate; IMP, inosine monophosphate; GMP, guanine monophosphate; PNP, purine nucleoside phosphorylase; XO, xanthine-oxidase; THF, tetrahydrofolate; FDA, Food and Drug Administration; GPCRs, G protein-coupled receptors; Hsp90, Heat shock protein 90; NSCLC, non-small-cell lung cancer.
